# Digestibility and transcriptomic profiling of kidney, pancreas, follicle, and uterus in laying hens supplemented with phytogenic feed additive

**DOI:** 10.1016/j.psj.2025.106219

**Published:** 2025-12-08

**Authors:** Henry Reyer, Michael Oster, Jürgen Zentek, Klaus Männer, Nares Trakooljul, Tobias Aumiller, Klaus Wimmers

**Affiliations:** a Research Institute for Farm Animal Biology (FBN), Wilhelm-Stahl-Allee 2, 18196 Dummerstorf, Germany; b Free University of Berlin, Department of Veterinary Medicine, Institute of Animal Nutrition, 14195 Berlin, Germany; c Delacon Biotechnik GmbH, 4209 Engerwitzdorf, Austria; d Faculty of Agricultural, Civil and Environmental Engineering, University Rostock, Justus-von-Liebig-Weg 6, 18059 Rostock, Germany

**Keywords:** Chicken, Essential oil, Gene expression, Saponin, thyme

## Abstract

Phytogenic feed additives in poultry demonstrate phenotypic improvements and potentially possess humoral significance for organ systems involved in egg production, digestion, and excretion, warranting further investigation into their mechanisms of action. These additives contain a wide array of active ingredients with antimicrobial, immunomodulatory, and antioxidant properties, positioning them as a promising alternative to in-feed antibiotics for enhancing poultry performance and welfare. This study investigates how a phytogenic feed supplement based mainly on essential oils of thyme and star anise with quillaja bark influences reproductive and nutrient-utilizing tissues and nutrient bioavailability in laying hens. A four-week trial with Lohmann Brown hens aged 68 weeks was conducted using diets with or without 150 mg/kg phytogenic supplement to assess performance (*n* = 96), egg quality (*n* = 96), nutrient digestibility (*n* = 24), and gene expression in pancreas, kidney, uterus, and follicles (*n* = 20 per tissue). Supplemented laying hens showed a significant increase of 3.84 % (*p* = 0.001) in the apparent ileal digestibility of crude protein and trends for improved egg weights (*p* = 0.053) and feed-to-egg mass ratio (*p* = 0.079) compared to control-fed hens. Complementary RNA sequencing analyses revealed differentially expressed genes (adjusted *p*-value < 0.05) with antimicrobial properties (pancreas, follicles) and an influence on bio-mineralization processes (uterus). Results suggest improved cleavage of carbohydrates, peptides, and lipids in the follicle, indicating improved nutrient utilisation for the developing embryo. Consequently, the implementation of phytogenics improves nutrient digestibility of laying hens and promotes resource reallocation in the hen towards the immune system and nutrient availability in eggs, potentially benefiting both *in-ovo* development and egg quality. A targeted phytogenic feed supplement can enhance the expression of antibacterial proteins involved in innate immune responses.

## Introduction

With the ban on using antibiotics as growth promoters for livestock species in the European Union (**EU**) and the public demand for reductions of antibiotic use in farm animal husbandry, a variety of feed additives such as probiotics, prebiotics, organic compounds with acidic properties, and phytogenics have been tested (Erkoc et al., 2024; Straus and Hancock, 2006). Phytogenic feed additives are a mixture of plant ingredients added to the feed with the aim to exploit the beneficial properties of the individual ingredients as well as the synergies between the components (Fernandes et al., 2025). For a phytogenic feed additive, ingredients may include, but are not limited to, essential oils, bitter compounds, saponins, flavonoids, mucilages, and tannins. These ingredients can increase appetite, improve feed properties, act as antioxidants and possess different capacities of antimicrobial action (Papatsiros et al., 2013). For poultry, various phytogenics are established to replace the use of commercial antibiotics to increase growth and laying performance, feed efficiency and immunocompetence (Kikusato, 2021; Masoud-Moghaddam et al., 2021; Shen et al., 2020).

The phytogenic formulation Biostrong® 510 (Delacon Biotechnik GmbH, Engerwitzdorf, Austria) is based on essential oils of thyme (*Thymus vulgaris*), star anise (*Illicium verum*), quillaja bark (*Quillaja saponaria*) as well as an array of herbs and spices (Bampidis et al., 2023). In broiler chickens, the addition of Biostrong® 510 to the feed was shown to improve growth rate, meat production and feed conversion (Lavrentyev et al., 2019). In laying hens, the supplementation of Biostrong® 510 has been associated with a slightly improved egg laying rate compared to control groups, suggesting a positive impact on reproductive performance (Bampidis et al., 2023). However, the complexity in the composition of phytogenic feed additives, the variability in the concentration of active compounds, and the formation of intermediate metabolites through biotransformation complicate the understanding of their specific mode of action. The efficacy of phytogenic feed additives depends on various farm-specific conditions related to housing, feeding, and genetics, and their practical application tends to be empirical rather than evidence-based. Current knowledge indicates that phytogenic additives can exert beneficial effects through multiple pathways, including antimicrobial, immunomodulatory, and antioxidant activities (Abdelli et al., 2021; Chu et al., 2025; Oso et al., 2019; Reyer et al., 2017). Indeed, dietary thymol supplementation has been shown to enhance egg weight and antioxidative capacity in serum of laying hens (Abd El-Hack and Alagawany, 2015), whereas others have reported unaltered production traits but improved polyunsaturated fatty acids (**PUFA**) levels in quail egg yolk (Fernandez et al., 2019). Moreover, *trans*-anethole has been characterized for its antimicrobial (Hançer Aydemir et al., 2018) and anti-inflammatory properties (Kim et al., 2017). However, the precise mechanisms by which these feed additives operate in livestock remain to be fully elucidated, necessitating further research to establish standardized protocols and confirm efficacy under varying management conditions. Holistic transcriptome analysis facilitates the hypothesis-free identification of gene expression alterations in tissues of interest, providing insights into the underlying mechanisms of action.

It is hypothesized that the use of phytogenic feed additives has a humoral significance for the organ systems related to egg production, the digestive system and excretion. This study aims to investigate the transcriptional effects of Biostrong® 510, a phytogenic feed additive, on reproductive and nutrient-utilizing tissues, particularly in the uterus, ovarian follicles, pancreas, and kidneys of laying hens. In addition, the bioavailability of nutrients was assessed by determining the apparent ileal digestibility.

## Materials and methods

### Ethics and consent to participate

The animal study was conducted in accordance with national guidelines and was approved by the Landesamt für Gesundheit und Soziales Berlin (A 0439/17). All procedures were in accordance with EU Directive 2010/63/EU on the protection of animals used for scientific purposes. The animal trial was performed at the Agricultural Experimental Station of the Free University of Berlin, Germany.

### Birds, housing, and diets

The hen trial was carried out with *n* = 96 birds from the high-yielding Lohmann Brown strain. At an age of approximately 68 weeks of life, hens were randomly assigned to one of two dietary groups. The trial design included 12 pens per dietary group, with each pen housing 4 birds. The experimental diets were fed for a period of 4 weeks and are display in Table 1. The diets contained either no phytogenic feed additives (control group) or were supplemented with 150 mg/kg Biostrong® 510 (**BS** group; Delacon Biotechnik GmbH, Steyregg, Austria). It was concluded that Biostrong® 510 is safe for chickens including layer lines (EFSA Panel, 2016). Zootechnical parameters (body weight, feed intake, egg production, egg weight, egg mass, feed-to-egg mass ratio), eggshell stability and egg yolk color (color fan) were recorded. After 4 weeks on trial, egg quality was assessed by determining Haugh units (calculated from albumen height and egg weight) and yolk index (ratio of yolk height to width). Eggshell breaking strength was measured on intact eggs using a compression apparatus that applied force at the equator between two flat plates. The force required to break the shell was expressed in Newtons (N). Egg nutrient composition was analysed on pooled yolk and albumen samples. Parameters included dry matter, crude protein, crude fat, crude ash, calcium, phosphorus, and amino acid profile. Analyses followed VDLUFA methods (dry matter: VDLUFA III 3.1; crude protein: VDLUFA III 4.1.1 modified for macro-N determination; crude fat: VDLUFA III 5.1.1; crude ash: VDLUFA III 8.1; calcium and phosphorus: VDLUFA VII 2.2.2.6). Amino acids were determined after acid hydrolysis using ion-exchange chromatography (VDLUFA III 4.11).

**Table 1:** Feed composition and calculated analysis of experimental diets.

Ingredients	Unit	Control diet	Biostrong® diet
Corn	%	67.54	67.54
Soybean meal (49 % CP)	%	20.50	20.50
Limestone	%	8.66	8.66
Soybean oil	%	0.84	0.84
Minerals and vitamins^1^	%	1.20	1.20
Dicalcium phosphate	%	1.02	1.02
DL- Methionine	%	0.10	0.10
L-Tryptophan	%	0.04	0.04
Wheat semolina	%	0.10	-
BIOSTRONG® *510* premix^2^	%	-	0.10
Marker (TiO_2_)	%	0.30	0.30
*Calculated nutrients*			
Moisture	%	10-12	10-12
ME^3^	MJ/kg	11.55	11.55
Crude protein	%	16.50	16.50
Lysine	%	0.81	0.81
Methionine	%	0.36	0.36
Methionine + Cystine	%	0.65	0.65
Threonine	%	0.63	0.63
Tryptophan	%	0.20	0.20
Crude fiber	%	2.22	2.22
Crude fat	%	3.92	3.92
Calcium	%	3.60	3.60
Phosphorus	%	0.50	0.50
Sodium	%	0.17	0.17

1 Contents per kg premix: 600000 I.U. Vit. A (acetate); 120000 I.U. Vit. D_3_; 6000 mg Vit. E (a-tocopherol acetate); 200 mg Vit. K_3_ (MSB); 250 mg Vit. B_1_ (mononitrate); 420 mg Vit. B_2_ (cryst. riboflavin); 300 mg Vit. B_6_ (pyridoxin-HCl); 1500 mg Vit. B_12_; 3000 mg niacin (niacinamide); 12500 mg biotin (commercial, feed grade); 100 mg folic acid (cryst., commercial, feed grade); 1000 mg pantothenic acid (Ca d-pantothenate); 60000 mg choline (chloride); 5000 mg iron (iron carbonate); 5000 mg zinc (zinc sulfate); 6000 mg manganese manganese oxide); 1000 mg copper (copper oxide); 45 mg iodine (calcium-iodate); 20 mg selenium (sodium-selenite); 140 g sodium (NaCl); 55 g magnesium (magnesium sulfate); carrier: calcium carbonate (calcium min 38 %); 0.25 g ß-apo-carotinoide acid-ester; 0.33 g canthaxanthine;

2 Contents per kg premix: 150 g Biostrong® 510, 850 g wheat bran;

3 Metabolizable energy (poultry).

### Sampling and analysis of ileal digesta

Apparent ileal digestibility of crude protein, amino acids, crude fat, crude ash, calcium, and phosphorus were determined in 12 birds (1 bird per replicate pen) of each treatment group selected for body weights closest to the average of the corresponding dietary group after 4 weeks on trial. For this purpose, birds were killed by exsanguination after mechanical stunning, about 3 hours after starting the lighting cycle. The posterior half between Meckels’ diverticulum and 3 cm cranial to the *osteum ileocaecale* was restricted. Ileal digesta was collected by purging the posterior ileum with a defined amount (5 ml) of water. The ileal contents of two birds each per treatment group were pooled (6 pooled samples per treatment) and samples were stored at - 20°C until freeze-drying and chemical analyses. Titanium (IV)-oxide (**TiO_2_**) was used as an inert marker at the dose level of 3 g/kg diet. Analysis of nutrients were in accordance to the methods issued by VDLUFA (dry matter: VDLUFA III 3.1; crude protein: VDLUFA III 4.1.1 modified according to macro-N determination (vario Max CN); crude fibre: VDLUFA III 6.1.4; crude ash: VDLUFA III 8.1; crude fat (ether extract): VDLUFA III 5.1.1; starch: VDLUFA III 7.2.1; total sugars: VDLUFA III 7.1.1; calcium: VDLUFA VII 2.2.2.6; phosphorus: VDLUFA VII 2.2.2.6; sodium: VDLUFA VII 2.2.2.6).

### Tissue sampling for transcriptomic analysis

In total, 10 representative animals of each dietary group were sampled between 0900 h and 1200 h at day 28 of the feeding trial. Therefore, birds were mechanically stunned and subsequently slaughtered by decapitation. The sampling included kidney tissue from the right half of the body, pancreas tissue taken in anatomical proximity to the duodenal loop, uterus mucosa collected 2-5 cm upstream of the cloaca, and 5-10 follicles with an average diameter of 3 to 5 mm, i.e., large white follicles. After dissection, the pancreas and uterus were rinsed with saline solution. All samples were snap frozen in liquid nitrogen and stored at −80°C. Individual oviposition was recorded while dissection. Specifically, the egg with the most advanced position in the oviduct was documented.

### RNA extraction and sequencing

For each of the four tissues, RNA was extracted from 20 samples (10 control vs. 10 BS per tissue). RNA preparation comprised an initial extraction using TRIzol Reagent (Invitrogen, Karlsruhe, Germany), a DNase I digestion (Roche, Mannheim, Germany) and a final clean-up using the RNeasy Mini Spin kit (Qiagen, Hilden, Germany). Total RNA of 2 µg was used for library preparation with the TruSeq Stranded mRNA Kit according to the manufacturer's recommendation (Illumina, San Diego, CA, USA). The sample-specific labelled libraries were multiplexed and sequenced for 2 × 71 bp paired-end reads in high-output mode on the Illumina HiSeq 2500. The raw data are available in the EMBL-EBI (www.ebi.ac.uk/arrayexpress) database under accession number E-MTAB-14922.

### Sequencing data processing and gene expression analysis

The initial quality check of the raw sequencing reads was performed with FastQC (version 0.11.8; https://www.bioinformatics.babraham.ac.uk/projects/fastqc/). By using Trim Galore (version 0.5.0; https://www.bioinformatics.babraham.ac.uk/projects/), reads of low quality (a mean Q-score < 20) and short reads (<30 bp) were filtered out and adapter-like sequences were trimmed. Mapping of the remaining reads to the chicken genome assembly (GRCg6a, Ensembl release 99) was performed using Hisat2 (version 2.2.0; https://daehwankimlab.github.io/hisat2/). Read counts that were uniquely assigned to each gene were extracted from the HISAT2 mapping results with HTseq (version 0.11.2).

Based on the annotated count data, the integrity of the data was initially checked in R (version 4.4.1) via hierarchical cluster analyses and principal component analyses, whereby all samples were classified as suitable for further processing (arrayQualityMetrics R package version 3.62.0; https://doi.org/10.18129/B9.bioc.arrayQualityMetrics). A principal component analysis (PCA) derived from RNA sequencing data of kidney, pancreas, uterus and follicle samples was conducted for dimensionality reduction and visualization of variance (arrayQualityMetrics R package).

### Statistical analysis

The performance data including bird and egg traits was analyzed by one-way ANOVA using SPSS software (IBM Corp. Released 2023. IBM SPSS Statistics for Windows, Version 29.0.2.0 Armonk, NY: IBM Corp). The individual hen or egg was the basic experimental unit. Apparent ileal digestibility data was analyzed by one-way ANOVA using SPSS. Data points were generated from 6 pooled samples per dietary group, with each pooled sample comprising data from 2 pens and thus representing 12 hens. Probability of *p* ≤ 0.05 was accepted as statistically significant, and *p*-values ranging from 0.05 to 0.10 were considered as trends. The identification of differentially expressed genes (**DEG**) between the experimental groups within the four tissues was performed using the package edgeR (version 4.4.2) in R (version 4.4.1), with each individual serving as a biological replicate (experimental unit) (Godfray et al., 2010). Genes that were expressed at very low levels in the dataset were excluded from further analysis using the filterByExpr function. Normalization followed the TMM algorithm implemented in edgeR, and DEG analysis was performed using a genewise negative binomial generalized linear model, considering the interaction of tissue and treatment group as fixed effects. Tissue-specific contrasts between the control and BS groups were obtained. Genes were considered as DEG with an adjusted *p*-value < 0.05. To identify the biological pathways represented by the DEG for each tissue, the lists of DEG were used for enrichment analyses with g:Profiler (Raudvere et al., 2019). The databases Gene Ontology (**GO**; Biological Processes), KEGG, and REACTOME served as resources. To focus on pathways with broader relevance, the analyses were restricted to terms with a maximum term size of 500 genes. Additionally, only terms with at least 3 DEG and a Bonferroni-adjusted *p*-value < 0.05 were considered significant. The enriched pathways were further clustered into a redundancy-free enrichment set for each tissue. GO terms were reduced based on semantic similarity, and KEGG/Reactome pathways were reduced based on gene set overlap (Jaccard index) using the R package simplifyEnrichment (version 2.0.0; Gu and Hübschmann, 2023). The term with the lowest *p*-value was indicated for each resulting cluster.

## Results and discussion

### Performance data

After 4 weeks on trial, no differences (*p* > 0.10) were observed on final body weight, body weight gain, or feed intake (Table 2). Birds selected for sampling of tissues also showed similar body weights at slaughter (mean ± SE; control birds: 1,775.5 ± 35.6 g; BS birds: 1,756.9 ± 66.6 g), which suggests that the phytogenic feed supplement had no effect on overall growth or body mass in this experimental setting. Recent studies have shown that the effects of phytogenic feed supplements on performance parameters, such as body weight and feed conversion ratio (FCR), depend on plant species, supplementation levels, and intrinsic animal factors such as growth stage as reviewed previously (Biswas and Kim, 2025). Regarding egg production parameters, egg weight (+1.4 %, *p* = 0.053) and feed-to-egg mass ratio (*p* = 0.079) showed tendencies to be improved by BS compared to the control hens. Other phytogenic feed supplements such as dried peppermint (*Mentha piperita* L.) leaves (Abdel-Wareth and Lohakare, 2014) and dried herbal powder containing thyme (Saki et al., 2014) have also been shown to positively affect egg weight and egg mass. In this study, the remaining parameters on laying performance or egg quality did not show statistically significant differences. Variations reported in the literature may result from differences in the type of phytogenic product, its application form, and management practices in reproductive poultry (Christaki et al., 2011; Abd El-Hack and Alagawany, 2015).

**Table 2:** Growth and laying performance from d 0 to d 28 on trial. Data represent means of 12 pens per dietary group with 4 hens/pen.

Item		Control diet	Biostrong® diet	*p*-value
Hens	n^o^	48	48	
Body weight – initial	g	1705.4 ± 71.9	1707.0 ± 51.0	0.950
Body weight – final	g	1762.6 ± 68.7	1758.2 ± 50.2	0.860
Body weight gain	g	57.2 ± 14.8	51.2 ± 10.4	0.264
Feed intake	g	3483.2 ± 148.6	3476.0 ± 182.5	0.916
Daily feed intake	g	124.4 ± 5.3	124.1 ± 6.5	0.916
Egg production	n^o^	19.7 ± 1.3	20.3 ± 1.7	0.282
Egg weight	g	64.1 ± 1.2	65.0 ± 0.9	0.053
Total egg mass	g	1257.8 ± 73.5	1322.1 ± 117.0	0.121
Feed-to-egg mass ratio		2.78 ± 0.19	2.64 ± 0.17	0.079
Breaking strength	10 x N	37.4 ± 3.8	37.8 ± 2.9	0.742
Yolk color^1^	Score	12.4 ± 0.4	12.5 ± 0.4	0.438

*p*-values of 0.05 < *p* ≤ 0.10 indicate a trend.

1 DSM Yolk color fan.

### Apparent ileal digestibility

The apparent ileal digestibility in layers was significantly affected by supplementation with BS for 28 days (Table 3) with crude protein (+5.7 %; *p* = 0.001), total amino acids (+3.1 %; *p* = 0.026), aspartic acid (+10.8 %; *p* = 0.022), calcium (+1.9 %; *p* = 0.041), and crude ash (+4.3 %; *p* = 0.013) being improved in comparison to controls. In addition, leucine (+6.4 %; *p* = 0.100) showed a tendency to be improved with BS compared to controls. Similar results were observed in broiler chickens when using a blend of essential oils from star anise, rosemary, thyme, and oregano in combination with *Quillaja saponaria* saponins as well as an essential oils mix (Reyer et al., 2017). It was suggested that the latter two components of the blend both have a positive effect on digestibility, although with different modes of action. Saponins are known to possess emulsifying properties affecting membrane permeability and nutrient absorption (Cheeke, 2000). The exact mechanisms by which essential oil compounds influence digestive processes are not fully understood. Their biological activity in the intestinal tract involves a variety of mechanisms, including modulation of the gut microbiome, antioxidant activity, interaction with intestinal receptors among others (de Sousa et al., 2023). Trans-anethole, and thymol, the main compounds of star anise, and thyme essential oils, respectively, are rapidly absorbed in the intestinal tract, potentially leading to systemic effects (Bampidis et al.; Ceylan et al., 2025; Van Noten et al., 2020). For trans-anethole, an effect on lipid metabolism was found in hepatocytes (Song et al., 2020) and the liver of broiler chickens (Yu et al., 2022), although both studies used higher doses than the present study. These liver-meditated metabolic effects can alter organismal lipid profiles, impacting intestinal function and potentially contributing to the observed improvement in apparent ileal protein.

**Table 3:** Apparent ileal digestibility of layers at d 28 on trial. Data represent means of 6 pooled samples of 12 hens per dietary group.

Items	Unit	Control diet	Biostrong® diet	*p*-value
Pooled replicates	n^o^	6	6	
Body weight	g	1758.0 ± 42.5	1752.3 ± 60.6	0.855
Dry matter (ileum digesta)	%	59.47 ± 4.23	59.74 ± 3.65	0.906
*Apparent ileal digestibility*				
Crude protein	%	67.84 ± 1.53	71.68 ± 1.40	0.001
- Alanine	%	69.58 ± 3.18	72.32 ± 3.66	0.197
- Arginine	%	79.36 ± 2.40	80.79 ± 2.35	0.322
- Aspartic acid	%	56.13 ± 5.15	62.21 ± 2.00	0.022
- Cysteine	%	80.33 ± 3.61	82.29 ± 3.27	0.347
- Glutamic acid	%	77.47 ± 1.87	79.02 ± 1.78	0.171
- Glycine	%	53.45 ± 8.34	57.77 ± 3.85	0.277
- Histidine	%	72.79 ± 2.79	74.00 ± 3.77	0.542
- Isoleucine	%	67.32 ± 3.41	66.92 ± 3.94	0.856
- Leucine	%	71.97 ± 3.05	76.60 ± 5.46	0.100
- Lysine	%	89.20 ± 1.99	90.08 ± 1.33	0.389
- Methionine	%	88.68 ± 1.82	90.30 ± 2.06	0.179
- Phenylalanine	%	75.52 ± 2.71	76.94 ± 2.47	0.366
- Proline	%	72.67 ± 2.11	73.89 ± 3.48	0.478
- Serine	%	66.76 ± 5.93	69.53 ± 2.03	0.304
- Threonine	%	63.97 ± 3.57	65.26 ± 3.45	0.540
- Tyrosine	%	73.35 ± 3.62	74.19 ± 5.58	0.763
- Valine	%	46.81 ± 4.90	50.78 ± 2.91	0.118
- Total amino acids	%	70.40 ± 1.74	72.59 ± 1.09	0.026
Crude fat	%	79.80 ± 1.06	80.20 ± 1.23	0.567
Crude ash	%	57.62 ± 1.86	60.07 ± 0.75	0.013
Calcium	%	63.23 ± 1.03	64.40 ± 0.66	0.041
Phosphorus	%	54.21 ± 2.96	56.51 ± 2.50	0.177

*p*-values of *p* < 0.05 indicate statistical significance and of 0.05 < *p* ≤ 0.10 indicate a trend.

### Transcriptional profiling

**Fig. 1:**
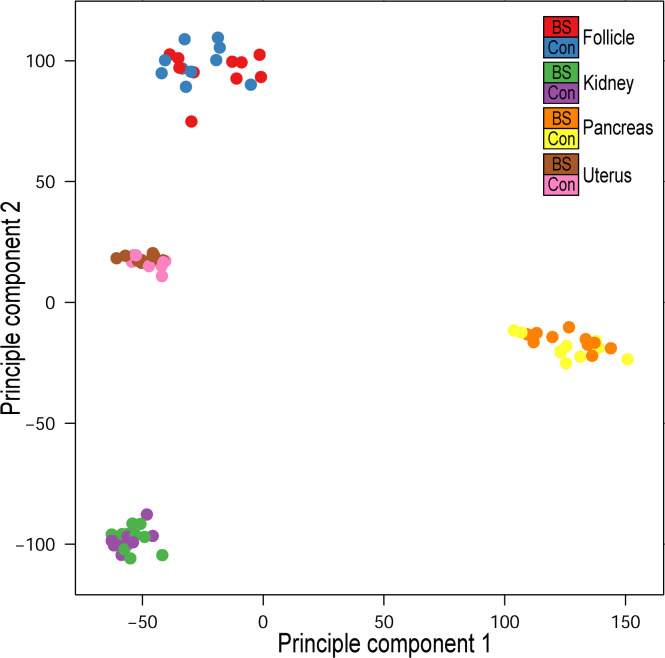
Principal component (PC) plot representing PC1 and PC2 derived from RNA sequencing data of kidney, pancreas, uterus, and follicle samples from laying hens fed either the standard feeding regimen (control) or supplemented with Biostrong® 510 (BS).

The RNA sequencing yielded an average of 25.8 million sequencing reads (± 4.9 million) per sample, with an overall alignment rate to the chicken reference genome of 96.7 % (± 1.0 %). The principal component (**PC**) plot represents the first two components of the RNA sequencing data of kidney, pancreas, uterus, and follicle samples from laying hens (Fig. 1) with clear separation of the distinct tissues. PC1 explained 32.7 % of the total variance while PC2 explained 29.1 % of the variance. PC 1 indicated the separation of the pancreatic expression profiles from the 3 other tissues, which were separated by PC 2. The secretory properties of the avian uterus, which is at the same time under endocrine control, is reflected by its position among the follicles and the kidney samples in the PC plot. All tissue-specific results of the differential expression analysis are shown in Supplementary Table S1.

For the comparison of gene expression profiles of kidney between control and BS fed hens, one gene was differentially expressed considering the significance threshold. This subtle effect on renal gene expression may be due to the primary effect of BS supplementation, which is to improve nutrient utilisation rather than affecting renal function of excretion and maintenance of mineral homeostasis. Renal *SLC25A25* was more abundant in BS treated hens compared to controls and represents a calcium-dependent mitochondrial transport protein. In murine models, targeted disruption of the *Slc25a25* gene resulted in diminished metabolic efficiency (Anunciado-Koza et al., 2011). These findings suggest that the upregulation of *SLC25A25* observed in kidney of BS hens may facilitate enhanced mitochondrial import of ATP and magnesium ions, thereby contributing to improved mitochondrial oxidative phosphorylation and ATP production.

In pancreas, 17 genes were found to be differentially expressed between the treatment groups. Besides a set of small RNAs, which mainly mediate regulatory actions, the DEG comprise *DPT* (Dermatopontin), *EPX* (Eosinophil Peroxidase), *S100A12* (S100 calcium-binding protein A12), *ATF3* (Activating Transcription Factor 3), *AvBD1* (Avian beta-defensin 1), *USP2* (Ubiquitin Specific Peptidase 2), and *HBE1* (Hemoglobin Subunit Epsilon 1). All the mentioned genes were more abundant in BS than in control hens. The pancreas, in addition to its exocrine functions of enzyme production for digestion and endocrine functions of hormone secretion for blood glucose regulation, also exhibits features related to immune response. The gene *ATF3* acts as a negative feedback regulator in inflammatory pathways, potentially providing protective effects against excessive inflammation (Kwon et al., 2015). The defensins AvBD1 and HBE1 are components of the innate immune system and are known for their antimicrobial properties (Cadwell et al., 2017). Upregulation of these defensins may enhance the host's defense against pathogens, contributing to the overall immune response. The gene DPT is involved in extracellular matrix (**ECM**) remodeling and may play a role in wound healing and tissue repair processes within the pancreas (Li et al., 2021). Taken together, results suggest that the increased nutrient availability demonstrated by the BS supplementation could support immune functions and stress resilience.

Comparing the uterine samples between the hens of the two groups, 496 DEG were identified. Out of these uterine-specific DEG, 94 genes were more abundant in BS than in the control group, while the majority of 402 DEG were more abundant in control hens than in BS hens. A potential explanation for this high amount of DEG compared to the other tissue types could be related to the reported estrogenic activity of trans-anethole by interacting with estrogen receptor alpha (ERα) as reviewed recently (Raposo et al., 2024). The highest significant DEG in the uterus were *RTBDN* (Retbindin), *ANO1* (Anoctamin 1), and *CREM* (cAMP Response Element Modulator). RTBDN, a key player in retinoic acid signaling, is essential for uterine receptivity and embryo implantation in mammals (Yin et al., 2021), whereas ANO1 may influence uterine contractility and ion/fluid transport (Jang and Oh, 2014). Additionally, CREM is involved in hormonal pathways critical for preparing the uterus for implantation (Bailey et al., 2000). The elevated expression of these genes in the control samples suggests enhanced uterine receptivity compared to the BS group. However, this finding contradicts the higher laying rate observed in the Biostrong® 510 group (Bampidis et al., 2023). The advanced age of the laying hens and the functional characterizations of the aforementioned genes derived from studies on mammals could partly explain this discrepancy. Downstream analysis of DEG revealed an enrichment in intracellular signaling pathways (MAPK signaling) as well as biological processes involving the MAPK and ERK1/ERK2 cascades (Table 4; Supplementary Table S2), which might be a direct response to the numerically higher apparent ileal digestibility of leucine in BS hens compared to controls (*p* = 0.100). The chicken uterus exhibits a distinct profile of genes that change dynamically throughout the laying period and are involved in eggshell formation through regulation of the extracellular matrix, mineral supply, and bio-mineralization pathways (Sun et al., 2023). In this study, a number of DEG were identified in the uterus that are enriched in the skeletal system development pathway. In the context of the uterus, this enrichment likely reflects processes related to bio-mineralization and eggshell formation. Here, the expression of *FAM20A* (FAM20A Golgi Associated Secretory Pathway Pseudokinase), *CCN1* (Cellular Communication Network Factor 1), and *BMP4* (Bone Morphogenetic Protein 4) was significantly lower in BS fed hens, whereas the expression of *KL* (Klotho) was significantly higher in BS fed hens compared to controls. It is conceivable that the downregulation of the former genes is linked to the more efficient nutrient absorption in BS supplemented animals compared to controls, resulting in an improved nutrient availability for post-absorptive tissues. The expression of *KL* in the avian uterus might be attributed to its role as a critical regulator in reproductive processes and calcium homeostasis as described in humans (Kanbay et al., 2024). The elevated expression of *KL* may account for increased mineral transport and extracellular matrix regulation, possibly enhancing uterine receptivity and functionality during critical periods of egg formation in BS supplemented hens. Potentially this change is related to the observed increase in calcium digestibility of the BS treated layer hens.

**Table 4:** Redundancy-free enrichment sets of follicle, uterus, and pancreas from laying hens fed either the standard feeding regimen (control) or supplemented with Biostrong® 510 (BS).

Term name	*p-*value	Genes
**Follicle**		
Ascorbate and aldarate metabolism (KEGG)	<0.001	*ENSGALG00000016570,MIOX,RGN,UGT1A1*
Carboxylic acid metabolic process (GO)	0.020	*ACSM3,ENSGALG00000016570,ENSGALG00000034772,* *HOGA1,PCK1,RGN,UGT1A1,UPB1*
Degradation of the extracellular matrix (R)	0.020	*CDH1,CTRB2,ENSGALG00000044661*
Digestion (R)	<0.001	*AMY2A,CEL,PNLIPRP2*
**Uterus**		
Ameboidal-type cell migration (GO)	0.029	*BCAR1,BMP4,CAP1,DUSP10,ENPP2,EPB41L5,EPHA2,* *FERMT1,FGF1,FGF2,JUP,LGALS8,MET,NR4A1,PDCD6,* *PLK2,PPARD,PTPRG,RGCC,SPRED1,SRC,THBS1,WNT7A*
Cellular response to growth factor stimulus (GO)	<0.001	*ADAMTS7,BCAR1,BMP4,CIDEA,COL2A1,CTNNB1,CYR61,EGR1,ENSGALG00000037160,EPB41L5,EXT1,FERMT1,* *FGF1,FGF2,FOS,HPGD,ITGB6,KL,LDLRAD4,NEO1,NOV,NR4A1,NREP,NTRK3,PDCD6,PDE8A,PMEPA1,SMAD3,* *SORL1,SPRED1,SRC,THBS1,TMEM100,WNT7A*
Epithelial to mesenchymal transition (GO)	0.045	*BMP4,CTNNB1,EPB41L5,FGF1,FGF2,LDLRAD4,PDCD6,PPP2CA,RGCC,SMAD3,SPRED1,TBX3,TMEM100*
MAPK cascade (GO)	0.025	*ATF3,BMP4,CTNNB1,CYR61,DUSP10,EPHA2,EZR,F2RL1,FGB,FGF1,FGF2,KL,LPAR1,MAP2K6,MAP3K5,MYC,* *NFKB1,NOV,NOX4,NTRK3,PDE8A,RNF149,SMAD3,* *SORL1,SPRED1,SRC,THBS1,TIRAP,TRIB1,YWHAZ,ZNF622*
MAPK signaling pathway (KEGG)	0.015	*AREG,CACNA1C,CACNA2D3,DUSP10,DUSP5,EPHA2,* *EREG,FGF1,FGF2,FOS,HSPA2,JUND,KRAS,MAP2K6,* *MAP3K5,MET,MYC,NFATC1,NFKB1,NR4A1,VEGFA*
Positive regulation of transferase activity (GO)	0.045	*CTNNB1,CYR61,DGKZ,EPHA2,EREG,FGF1,FGF2,GINS2,LMO4,MAP2K6,MAP3K5,NOX4,NTRK3,PKD1,PPP2CA,* *RGCC,SORL1,SRC,TCIM,THBS1,TIRAP,TNFSF15,VLDLR,ZNF622*
Skeletal system development (GO)	0.021	*ADAMTS7,ARID5A,BMP4,COL13A1,COL2A1,CTNNB1,* *CYR61,EPHA2,EXT1,FGF2,FLVCR1,FMN1,HOXB4,ITGB6,MAP2K6,MCPH1,MIGA2,NOV,PIP4K2A,PKD1,PKDCC,* *SMAD3,SRC,TAPT1,TBX3,TTC9,WNT7A*
**Pancreas**		
Neutrophil degranulation (R)	0.035	*EPX,HBBA,S100A12*

R – Reactome database; *p*-value – Bonferroni-adjusted *p*-value.

The analysis of the follicle samples revealed 40 DEG considering the significance threshold. Interestingly, only *VTG2*, which encodes vitellogenin-2, was found more abundantly expressed in the control birds than in BS birds. The expression of vitellogenin genes in liver tissue, including *VTG2*, was positively influenced by probiotic supplementation in aged hens at 19 months of age and correlated with an increase in the number of formed follicles (Mazanko et al., 2019). The *VTG2* expression pattern might therefore not confirm a follicle-stimulating effect of the BS treatment, however, *VTG2* exhibited very low expression levels in both dietary groups as indicated by the logCPM levels (Supplementary Table S1). Among the DEG from the analyzed follicles, the potent antibacterial protein *LYG2* (lysozyme G2) showed a higher expression due to the phytogenic feed supplementation compared to the control hens. By enhancing the expression of *LYG2*, the phytogenic feed additives could support the innate immune response and might provide a more robust defense against microbial infections (Kovacs-Nolan et al., 2005; Salton, 1957). Additionally, transcripts encoding *AMY1B* (amylase alpha 1B), *CPB1* (carboxypeptidase B1), *CTRB1* (chymotrypsinogen B1), *CEL* (carboxyl ester lipase), *PNLIPRP1* (pancreatic lipase related protein 2), and *CLPS* (colipase) showed significantly elevated mRNA abundances in BS hens compared to controls. Indeed, these genes are known to be expressed in follicles of different stages, i.e., pre-hierarchical, pre-ovulatory, and post-ovulatory stages, and are therefore subject to a maturity-related dynamic (Kui et al., 2024). Since the above-mentioned genes are associated with the systematic cleavage of carbohydrates, peptides, and lipids, it is conceivable that their upregulation in the follicle supports the timely provision of essential amino acids, fatty acids, and carbohydrates for the growth of the developing embryo in the fertilized egg, potentially benefiting chick viability and hatch performance (Liu et al., 2021). In the context of egg production for human consumption, this would indicate an improved nutrient bioavailability of the egg through the BS supplementation (Lv et al., 2024). In follicles, the BS fed hens additionally exhibited increased expression of the sodium/phosphate cotransporter *SLC34A1* (solute carrier family 34 member 1) and the myo-inositol-degrading enzyme *MIOX* (myo-inositol oxygenase). The expression of *SLC34A1* has been shown to be tissue-specific and sensitive to nutritional regimens with high abundances in the avian kidney to follow metabolic demands (Omotoso et al., 2023). The increased expression of *MIOX* might lead to the formation of glucuronic acid as a precursor in the biosynthesis of ascorbic acid or to ensure biotransformation, i.e., glucuronidation (Table 4; Supplementary Table S2) (Hooper et al., 2000).

In conclusion, the usage of phytogenic feed additives is a promising strategy to improve nutrient digestibility of laying hens with potential benefits on egg mass and egg-to-feed ratio. The positive effect of the feed additive on apparent ileal digestibility is linked to altered resource allocation towards immune support and enhanced nutrient availability in eggs, potentially benefiting *in-ovo* development. The molecular analyses showed that both pancreas and follicles responded to BS supplementation with increased expression of genes with described antimicrobial properties, thereby generating immune support for the hen’s intestinal and reproductive tracts. Furthermore, the transcriptional patterns suggest that the mediated phytogenic effects culminated in the modulation of macronutrient metabolism (follicles) and bio-mineralization (uterus). Strategic phytogenic feed supplementation may have the potential to stimulate potent antibacterial proteins associated with innate immunity, which needs to be investigated in studies concerning challenged animals.

## CRediT authorship contribution statement

**Henry Reyer:** Writing – review & editing, Writing – original draft, Visualization, Methodology, Investigation, Formal analysis, Data curation. **Michael Oster:** Writing – review & editing, Writing – original draft, Investigation, Data curation. **Jürgen Zentek:** Writing – review & editing, Supervision, Resources, Methodology, Investigation, Funding acquisition, Data curation, Conceptualization. **Klaus Männer:** Writing – review & editing, Resources, Methodology, Investigation, Data curation, Conceptualization. **Nares Trakooljul:** Writing – review & editing, Methodology, Investigation. **Tobias Aumiller:** Writing – review & editing, Writing – original draft, Supervision, Resources, Project administration, Methodology, Investigation, Funding acquisition, Formal analysis, Data curation, Conceptualization. **Klaus Wimmers:** Writing – review & editing, Supervision, Resources, Investigation, Funding acquisition.

## Disclosures

The authors declare the following financial interests/personal relationships which may be considered as potential competing interests:

Tobias Aumiller is an employee of Delacon Biotechnik GmbH, a manufacturer for phytogenic feed additives, which is a wholly owned subsidiary of Cargill, Incorporated. If there are other authors, they declare that they have no known competing financial interests or personal relationships that could have appeared to influence the work reported in this paper.

## Appendix Supplementary materials

Supplemental Table S1. Differentially expressed genes comparing laying hens supplemented with Biostrong 510 (BS) versus controls. Image, application 1

Supplemental Table S2. Tissue-specific enrichment of pathways based on differentially expressed genes comparing laying hens supplemented with Biostrong 510 versus controls. Image, application 2
